# Conventional Gingivectomy Procedure in the Management of Orthodontic-Induced Gingival Overgrowth: A Case Report

**DOI:** 10.7759/cureus.64556

**Published:** 2024-07-15

**Authors:** Aakanksha M Dalal, Ranu R Oza, Unnati Shirbhate, Tikeshwari Gurav

**Affiliations:** 1 Department of Periodontics and Implantology, Sharad Pawar Dental College and Hospital, Datta Meghe Institute of Higher Education & Research, Wardha, IND; 2 Department of Endodontics, Sharad Pawar Dental College and Hospital, Datta Meghe Institute of Higher Education & Research, Wardha, IND

**Keywords:** orthodontic treatment, gingivoplasty, scalpel, gingivectomy, gingival enlargement

## Abstract

Gingival enlargement (GE) can result from gingival inflammation, fibrous overgrowth, or a combination of both factors. Various etiological factors contribute to GE, including low-grade trauma, iatrogenic causes, drug-induced effects, systemic diseases, plaque accumulation, hormonal influences, vitamin deficiencies, genetic predispositions, and idiopathic reasons. Effective treatment in clinical practice hinges on accurately diagnosing the underlying cause. Among these, plaque-induced inflammation is the most common, driven by the accumulation of plaque and calculus. One challenge in maintaining oral hygiene is orthodontic treatment, which can impact speech, chewing, aesthetics, and psychological well-being. In this case report, a 21-year-old female patient developed GE associated with orthodontic appliance use. To address this, excess gingival tissue was surgically removed under local anesthesia using gingivectomy and gingivoplasty procedures, and the excised tissue was sent for histopathological examination. Following the surgery, a periodontal dressing (GC Coe Pack™) was applied to protect the tissue and aid in healing. The case underscores that enlarged gingival tissue, covering nearly half of the dental crowns, led to plaque accumulation and aesthetic concerns. Post-procedure, achieving a proper gingival contour eliminated suprabony pockets and enhanced the aesthetic appearance. The patient showed positive outcomes with no remaining suprabony pockets, resulting in a natural gingival contour, improved aesthetics, and reduced plaque retention. Surgical gingivectomy and gingivoplasty proved to be successful interventions in this case.

## Introduction

The increase in size of the gingiva is referred to as gingival enlargement (GE), and it is a standard indicator of gingival disease. It is a condition brought on by interactions among the number of stimuli, the host, and the environment. GE, generally, results from inflammatory changes induced by the retention of bacterial dental plaque, which can occur locally or spread throughout the tooth, and less efficient oral hygiene care [1]. Throughout their orthodontic treatment, individuals experience GE due to a microbial assault and the host’s immune responses. Both characteristics must coexist in balance for periodontal disease to begin and spread in the orthodontic patient, resulting in the persistence of GE [2]. Patients who have used fixed orthodontic treatment for a period exceeding 12 months are likely to experience periodontal diseases during their orthodontic therapy. The persistence of bacterial dental plaque in individuals undergoing orthodontic treatment has been linked to periodontal issues [3]. They noticed gingival overgrowth or alterations in the tissue because orthodontic brackets consist of metal alloy components, like nickel, from which specific braces are constructed. Fibroblasts proliferate when exposed to interactions among nickel ions and gingival tissues inside the oral cavity and cause gingival overgrowth [4].

A gingivectomy is a surgical procedure that eliminates excess gingival tissue from the pocket wall. By performing that, plaque, calculus, and other irritants are eliminated, allowing visibility and accessibility. This process can predict an optimal environment for gingival healing and reconstruct the gingiva’s standard physiological shape [5]. Reshaping the gingiva into knife-edge borders is essential as an element of the gingivoplasty treatment. Gingivoplasty achieves a physiologic gingival contour and reconstructs the gingiva with pocket elimination. It is a procedure that can improve the aesthetics of the gingiva. A gingivectomy can be done by scalpel, electrosurgery (cautery), laser, or chemosurgery [6]. The patient’s periodontal health must be carefully assessed while receiving orthodontic treatment. The individual receiving treatment should be instructed to maintain good oral hygiene and informed about prevention, brushing techniques, controlling dental plaque, eliminating etiological variables, and indicating the patient’s required hygiene devices [7]. This article aims to report a clinical scenario utilizing the gingivectomy surgical procedure, which uses a scalpel to treat GE induced by orthodontics.

## Case presentation

**Figure 1:**
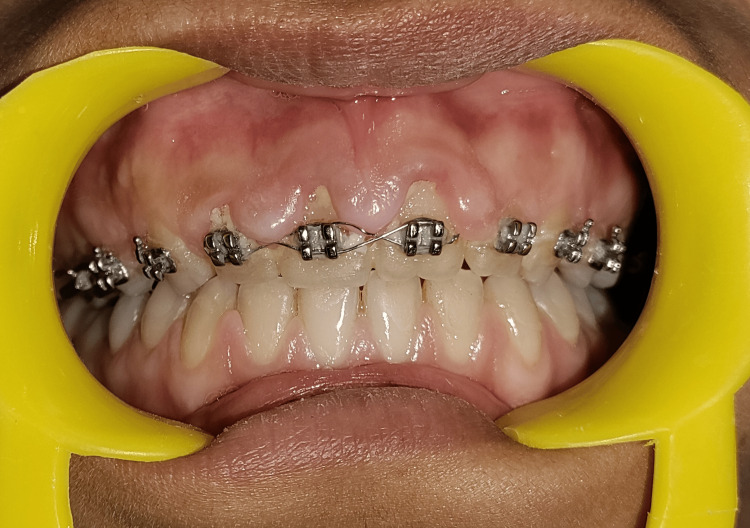
Preoperative image of the patient undergoing orthodontic treatment, illustrating GE encroaching upon the brackets GE, gingival enlargement

A 21-year-old female patient undergoing orthodontic treatment at Sharad Pawar Dental College and Hospital presented to the Department of Periodontics with complaints of swollen gums. Clinically, she exhibited marginal and interdental GEs in the upper anterior region, as depicted in Figure 1. The patient had been undergoing orthodontic therapy for one year. Subsequently, upon discovering the GE, it was noted that the patient had difficulty maintaining good dental hygiene.

The patient had no relevant medical or family history. The gingiva appeared pale pink, soft, and friable, with a rounded contour. Examination revealed extensive gingival tissue and pseudopockets with probing depths of 4-5 mm. Local factors such as calculus and plaque were present on every tooth. Based on these findings, the patient was advised to undergo initial therapy, surgical intervention, and a hematological evaluation.

Initially, supragingival scaling and polishing were performed to reduce fibrotic components, and the patient was instructed to use a soft toothbrush and practice gentle brushing, following the modified bass technique. Hematological tests, including hemoglobin level, clotting time, bleeding time, and random blood sugar, were within normal limits. A follow-up after seven days showed reduced bleeding and gingival inflammation, with the GE remaining soft and friable post-scaling.

**Figure 2:**
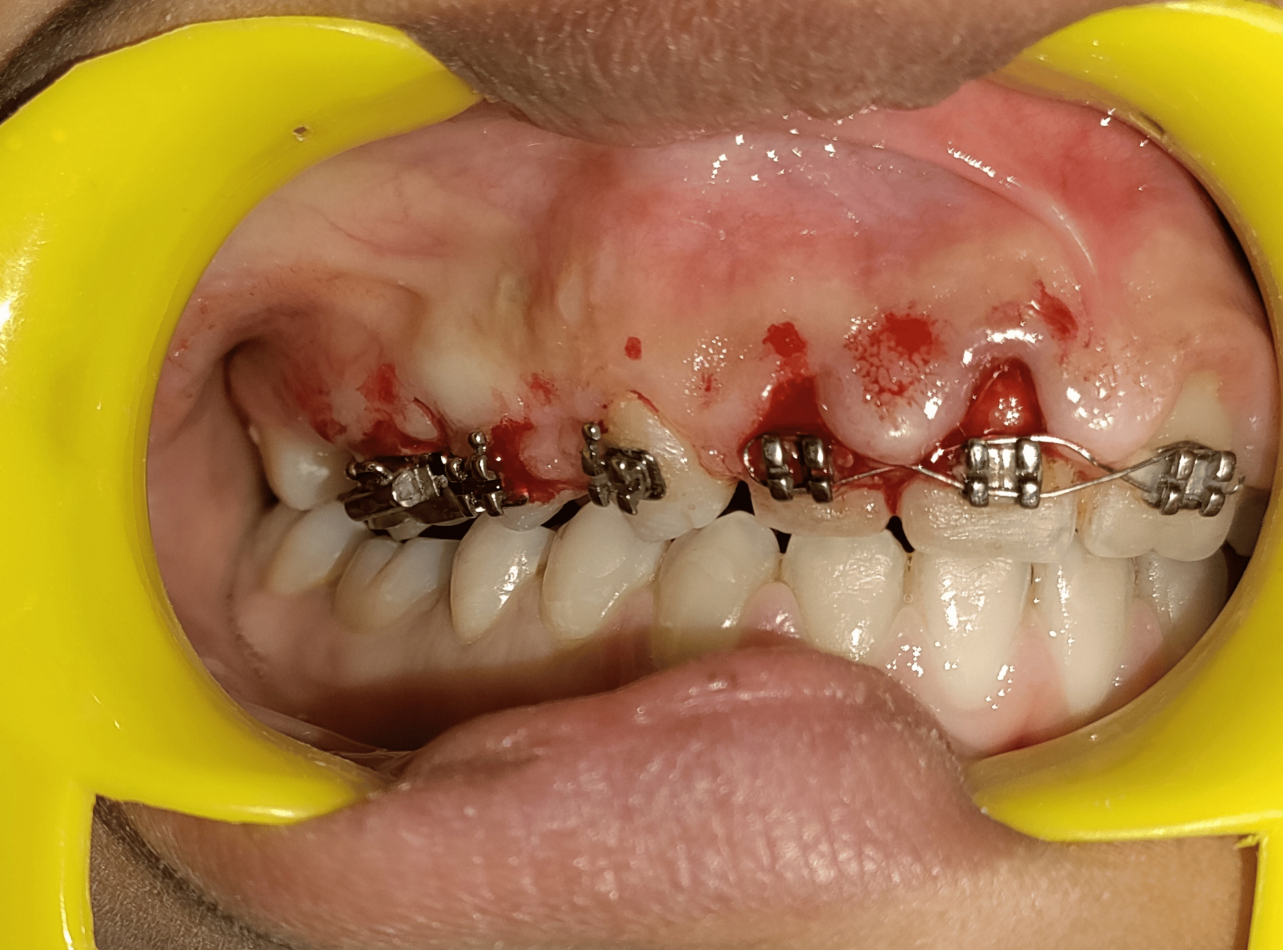
Bleeding points marked by the pocket marker corresponding to the depth of the pockets

Subsequently, a gingivectomy procedure was scheduled, with the patient providing informed written consent. Under local anesthesia (2% lidocaine with 1:80,000 epinephrine), a bilateral supraperiosteal nerve block was administered following standard aseptic protocols. A pocket marker was used to identify bleeding points, as depicted in Figure 2.

A gingivectomy was performed using the conventional scalpel method. External bevel incisions were made with a no. 15 BP blade, followed by a crevicular incision extending beyond the markings. An Orban knife #1/2 was utilized to release interdental tissue, and tissue tags were removed using a curette and scissors. Gingivoplasty was then conducted to achieve a smooth and physiologically contoured gingival surface with a scalpel. The excised tissue was sent for histopathological examination.

**Figure 3:**
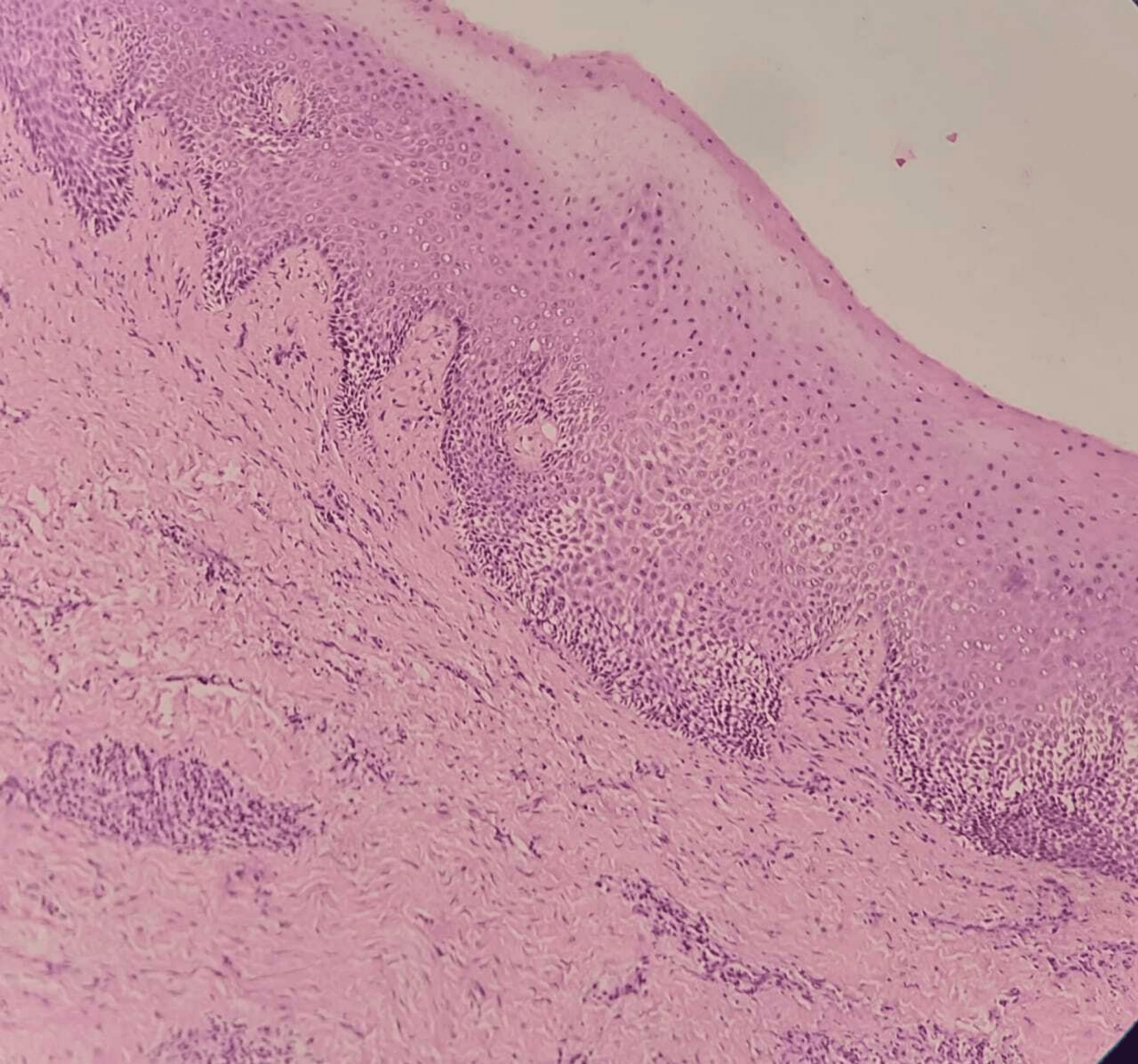
Histological section demonstrating hyperplastic parakeratinized epithelium and connective tissue infiltrated with chronic inflammatory cells

Under low-power microscopic view with hematoxylin and eosin staining, the histopathological section revealed proliferative hyperplastic stratified squamous parakeratinized epithelium overlying inflamed connective tissue. The underlying stromal tissue exhibited bundles of collagen fibers, plump and spindle-shaped fibroblasts, and focal aggregates of chronic inflammatory cells, predominantly plasma cells, lymphocytes, and blood vessels. Clinical-pathological correlation indicated inflammatory fibrous hyperplasia, as depicted in Figure 3.

**Figure 4:**
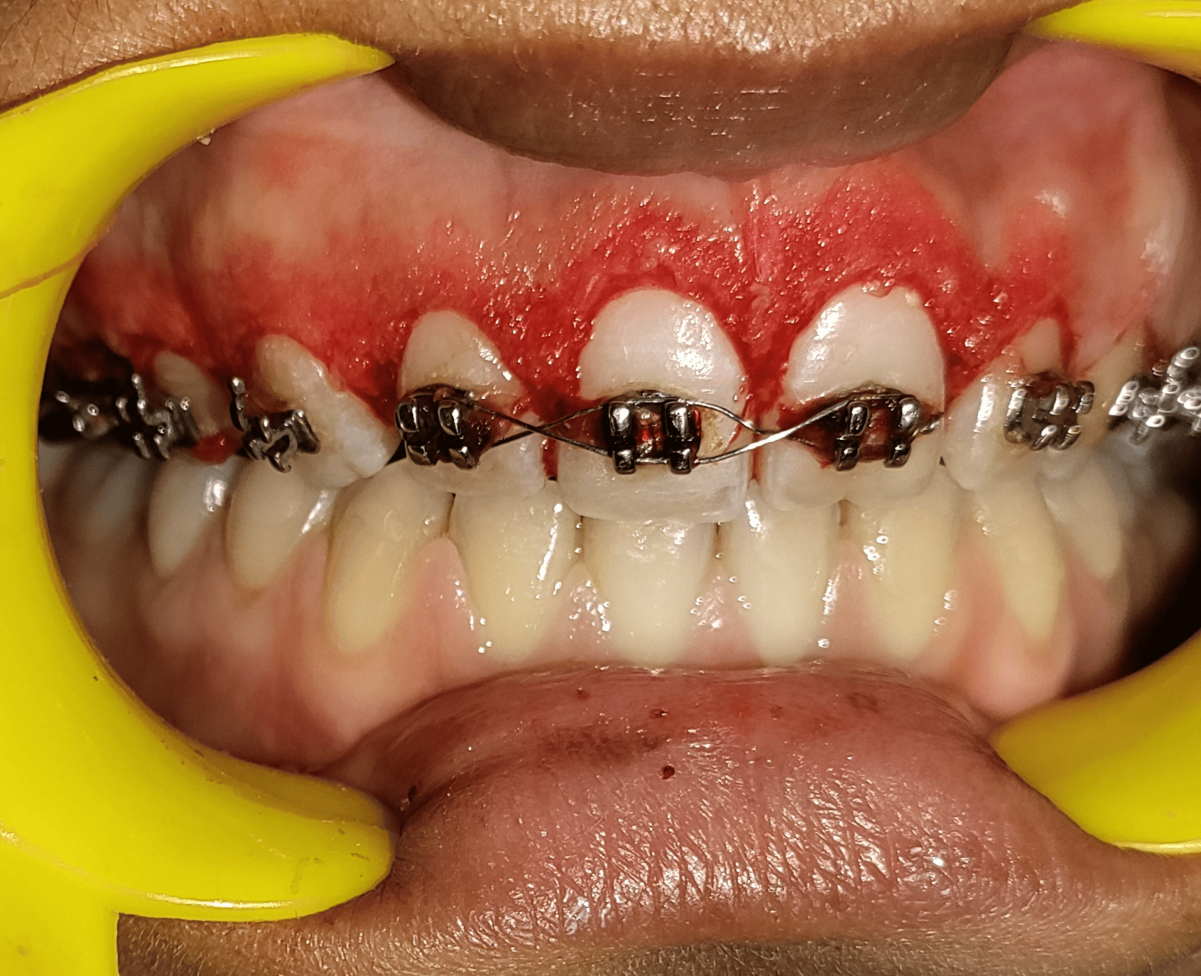
Gingivectomy and gingivoplasty performed using the conventional scalpel technique

The scalpel-based gingivectomy and gingivoplasty procedures resulted in a notable improvement in the orthodontic treatment related to GE, as shown in Figure 4.

**Figure 5:**
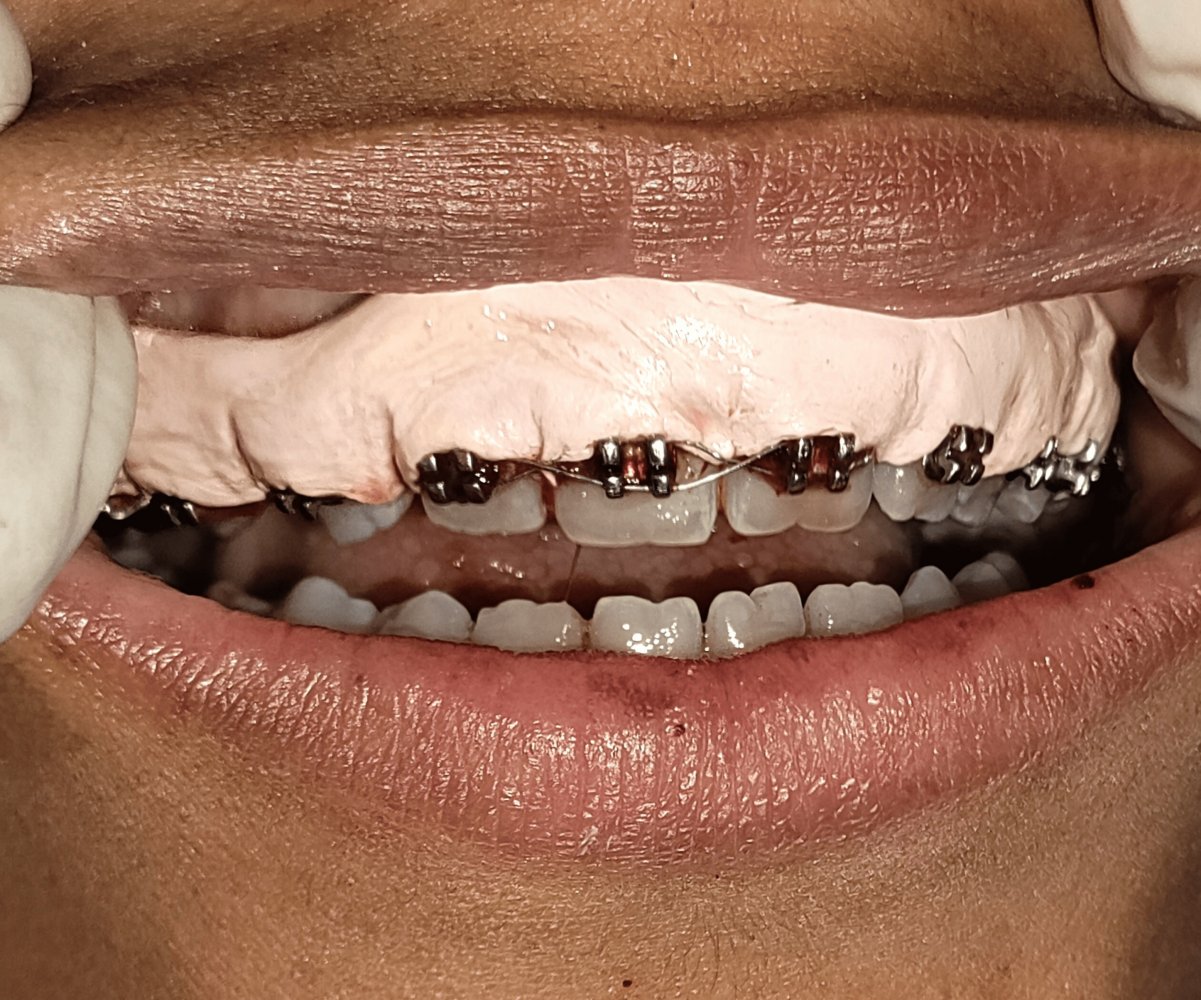
Periodontal dressing applied post-surgery

After achieving hemostasis, a periodontal dressing (GC Coe Pack™) was applied to protect the tissues and facilitate healing, as illustrated in Figure 5.

After the procedure, the patient received analgesics and an antiseptic mouth rinse, following postoperative instructions. Seven days later, the patient returned for the removal of the periodontal dressing. The achieved gingival contour was found to eliminate suprabony pockets, promote satisfactory healing, and enhance aesthetic appearance.

**Figure 6:**
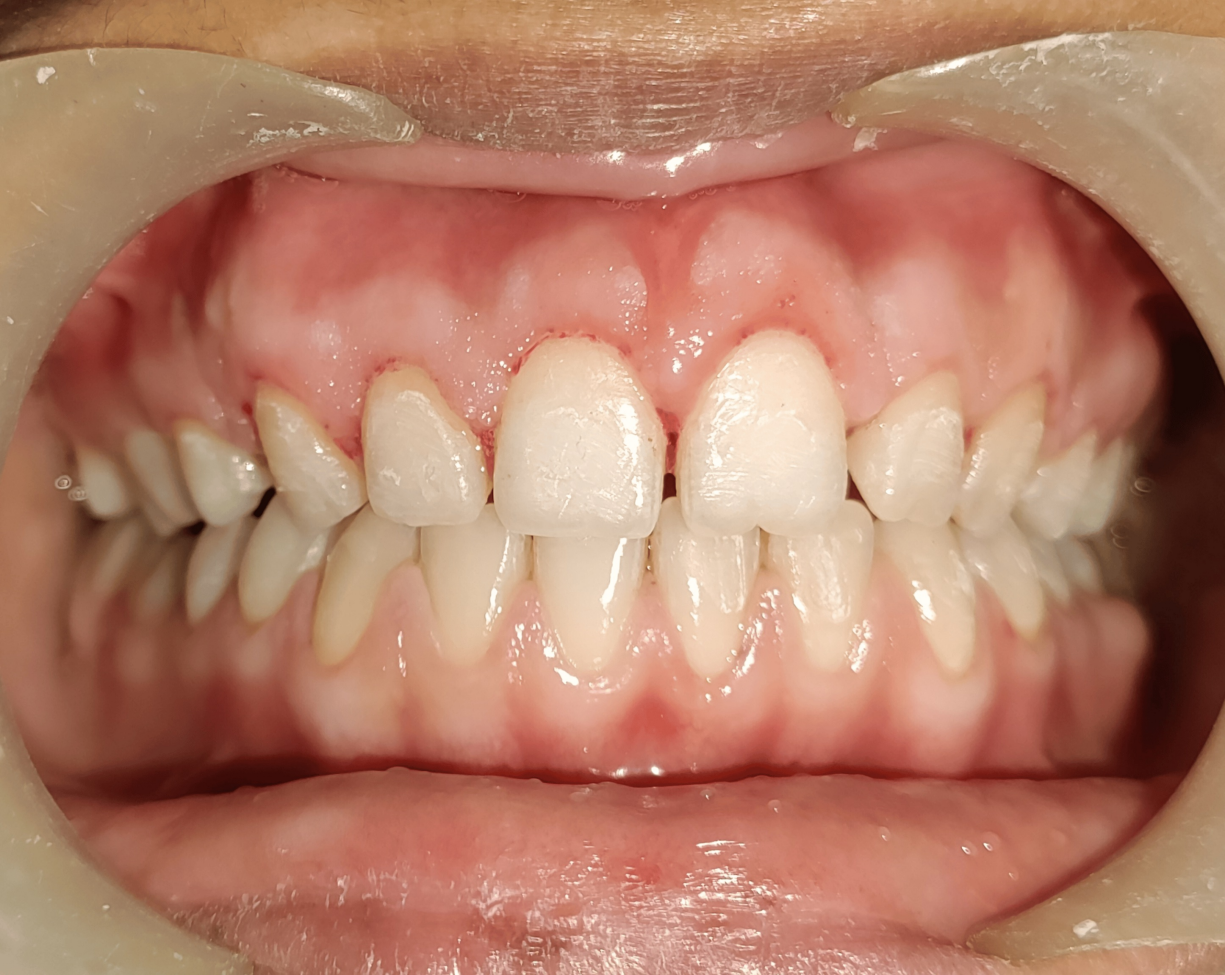
Two months post-surgery, a favorable outcome was observed during follow-up, demonstrating excellent clinical crown exposure

A follow-up examination at two months showed complete healing and favorable outcomes regarding gingival inflammation post-treatment, as depicted in Figure 6.

## Discussion

Orthodontic treatment is one factor that triggers gingival overgrowth or enlargement in patients. GE reported a prevalence of 10% in orthodontic patients. The enlargement can occur in a variety of ways [8]. GE can be localized or generalized and is caused mainly by inflammation due to plaque. Plaque stimulates the stay for longer, leading to chronic inflammation and the proliferation of fibrous connective tissue [9]. The fixed orthodontic treatment contributes to more plaque accumulation, which has been determined to be the primary cause of periodontal disease. When the orthodontic treatment lengthens, the frequency of GE increases. Orthodontic treatment participants are likelier to experience anterior gingival hypertrophy [10]. They also found an association between excess resin around brackets and proximal anterior gingival bleeding. To prevent GE, educational initiatives should be undertaken to achieve orthodontic therapy for gingival health. Surgical methods are required to treat gingival tissue inflammations that hinder prompt orthodontic finishing when oral hygiene care is still inadequate [2].

Gingivectomy and gingivoplasty establish harmonious gum lines, prevent GE, and sustain good oral hygiene. The gingivectomy technique is a simple method that most patients find acceptable, obtaining satisfactory results in aesthetics and harmony according to the correct indications [11]. Performing a gingivectomy with a scalpel offers several advantages. The technique is relatively straightforward, allowing precise incisions on the predetermined marginal gingiva. Additionally, healing is typically excellent and rapid compared to other methods. However, there are drawbacks to this technique, including the possibility of bleeding that occurs during the surgical procedure so that it interferes with the operator’s view. In addition, the presence of pain after surgery and the possibility of a prolonged healing process are factors that need to be considered [12]. The present case is regarded as a successful therapy when the chronic GE is corrected aesthetically by scalpel surgical gingivectomy and gingivoplasty.

## Conclusions

The most common side effect of orthodontic therapy is gingival overgrowth. It is crucial to conduct a thorough evaluation of the patient’s periodontal health and provide comprehensive education before initiating orthodontic treatment. This case highlights the beneficial impact of surgical periodontal therapy, specifically scalpel gingivectomy, on effectively managing gingival health issues for patients with fixed orthodontic appliances.

Gingivectomy and gingivoplasty procedures are shown to eliminate suprabony pockets, restore a natural gingival contour, and enhance the patient’s aesthetic satisfaction. These techniques involve additional effort but contribute significantly to achieving better outcomes, potentially reducing discomfort both during and after treatment.
